# On the Use of Short-Interfering Rna to Study Alcohol-Related Genes

**Published:** 2008

**Authors:** Christopher A. Adams, W. Michael Zawada

**Keywords:** Alcohol consumption, AOD effects and consequences, brain, brain function, genetic factors, DNA, RNA, short-interfering RNAs (siRNAs), silencing

One strategy to determine the contribution of individual genes to the development of complex traits and behaviors such as alcohol consumption is to reduce or completely eliminate the activity of those genes in the cells or organism under investigation and then study the effects of this modification. But how can a single specific gene be inactivated? One strategy that has been developed in recent years is the use of small, artificially generated molecules called short-interfering RNAs (siRNAs). This article briefly describes the principles of this strategy and presents some initial results obtained with this approach.

## Principles of siRNA

RNA is a naturally produced molecule in the body that is involved in the process of converting the genetic information encoded in DNA into protein products (i.e., in gene expression). Its structure is very similar to that of DNA. However, whereas DNA typically consists of two chains, or strands, of building blocks (i.e., nucleotides) sticking together, RNA normally is a single-stranded molecule. When a gene is expressed, the genetic information from one strand of DNA is first copied into a complementary RNA molecule known as messenger RNA (mRNA). The mRNA molecule then travels into a different region of the cell, where it serves as a template for the production of a protein molecule in a process called translation. However, if the mRNA is degraded before it can be used to produce protein molecules, the expression of the corresponding gene is prevented—a phenomenon known as silencing. siRNA does just that: It can interact with a specific, complementary mRNA, thereby inducing its degradation or at least repressing translation.

The discovery that short, double-stranded RNA molecules could be generated and processed into siRNA paved the way for the use of these molecules for silencing gene expression in mammals (Elbashir et al. 2001; Harborth et al. 2001). To use this approach, researchers must know the exact DNA sequence of the gene under investigation and create a double-stranded RNA molecule with the exact same sequence. These siRNA molecules consist of 21 to 23 nucleotide pairs of double-stranded RNA with a two-nucleotide overhang at the 3′ end of each strand. One of the strands is called the “guide” strand and the other one the “passenger” strand. The guide strand is the one that can bind to the target mRNA; together with other molecules, the mRNA– siRNA complex forms the so-called RNA-induced silencing complex (RISC), which then mediates degradation of the target mRNA (for an in-depth review of this and related processes, see Hutvagner and Simard 2008; Tomari and Zamore 2005). siRNA molecules that are completely complementary to the target mRNA sequence trigger the destruction of the target mRNA (Fire et al. 1998). If siRNA and target mRNA differ in one or two nucleotides, however, translation of mRNA into a protein is repressed but not completely abolished (Saxena et al. 2003).^1^

As indicated earlier, the siRNA approach can only be used if the exact DNA sequence of the target gene (and thus of its mRNA) is known, so that the corresponding siRNA molecules can be created in the laboratory. However, to be most effective, siRNA should not be targeted to just any region of mRNA because the stability of the mRNA– siRNA complex and the effectiveness of the resulting mRNA destruction differs depending on the specific nucleotide sequence of siRNA. Synthetic siRNA design is being continually refined and is based on a rational-design algorithm that takes into consideration eight characteristics associated with siRNA functionality (Reynolds et al. 2004). These refinements constitute a significant design improvement from the selection rules for siRNAs originally described by Elbashir and colleagues (2002). Today, most siRNA molecules can be ordered from a variety of commercial vendors, and a practical way to design the siRNAs needed for a given experiment is to utilize design algorithms available on vendors’ Web sites. Moreover, siRNAs should be designed in a way that limits effects on other mRNAs that show some complementarity to the siRNA molecule so that they are not inadvertently silenced as well (i.e., off-target effects).

Another important issue is how to bring the siRNA molecules into the target cells so that they can interact with the target mRNA. Although synthesized siRNA duplexes cannot passively cross the cell membrane, they can be introduced into cultured cells using a technique known as transfection. With this technique, siRNA molecules are introduced into the cells with the aid of fat-like molecules carrying a positive electrical charge (i.e., cationic lipids) or polymer nanoparticles (Zhang et al. 2007), and many manufacturers continue developing new lipids for siRNA delivery to improve its effectiveness. Cationic lipids also have been tested as a vehicle for intra-venous siRNA administration into a living organism (i.e., in vivo). However, this leads to the activation of other signaling molecules that result in the activation of various genes; therefore, this approach should be used with caution in vivo (Ma et al. 2005). Moreover, in the central nervous system (i.e., the brain) the presence of free lipids can cause considerable cytotoxicity. For these reasons, researchers currently are attempting to chemically modify siRNAs so that they can be transfected into cells without the use of lipids. Some investigators found that siRNA could be introduced effectively into cells if it was linked to cholesterol (Kubo et al. 2008). The cholesterol may increase siRNA stability by incorporating it into lipoprotein particles that protect siRNA from breakdown by cellular enzymes (i.e., nucleases) (de Fougerolles et al. 2007). Moreover, cholesterol increases uptake of the bound siRNA through the membrane into the cell (de Fougerolles et al. 2007; Wolfrum et al. 2007). Another strategy to deliver siRNA to specific cells is to combine it with complexes containing a molecule called protamine, which binds to siRNA, and immune molecules (i.e., antibodies) that specifically bind to molecules on the surface of the target cells. Such an approach was used to deliver siRNA to HIV-infected cells (Song et al. 2005).

## Effectiveness of siRNA Approaches

Several studies have demonstrated that the siRNA approach can be successfully used to reduce or eliminate expression of a specific gene. Even a single injection of siRNA into a specific brain region has been shown to be effective for some targets. For example, a single injection of siRNA intended to reduce expression of a molecule called preproorexin into a certain region of the rat hypothalamus reduced the prepro-orexin mRNA levels by 59 percent within 2 days of administration (Chen et al. 2006). Furthermore, mRNA levels for that molecule returned to their normal levels by the 5th day after the injection. Orexins are peptides best known for their role in regulating the sleep–wakefulness cycle, during which they promote wakefulness. The effectiveness of the aforementioned siRNA regimen in reducing mRNA levels and thereby preventing production of the corresponding peptide also was supported by the fact that in the animals which had received the treatment, the number of rapid eye movement episodes observed during their sleep cycles increased, indicating prolonged periods of sleep and reduced wakefulness (Chen et al. 2006).

Other studies have investigated the possibility of using siRNA in treating Huntington’s disease, which involves a protein called huntingtin. Huntingtin is a protein expressed in the brain and other tissues whose exact function is not known but which is thought to play an important role in signaling, transporting materials, binding proteins and other structures, and protecting against programmed cell death. The gene encoding this protein has a characteristic sequence of the three nucleotides, CAG, at one end. In healthy people, this sequence is repeated up to 35 times in a row. In people with Huntington’s disease, however, the gene carries more than 35 copies of this nucleotide sequence, resulting in an abnormal protein. To investigate the therapeutic silencing potential of the siRNA approach, researchers performed a single injection of siRNA to the huntingtin mRNA into the brain of mice expressing excessive levels of huntingtin protein containing 100 CAG repeats. This treatment prevented accumulation of the abnormal huntingtin protein (DiFiglia et al. 2007).

**Figure 11:**
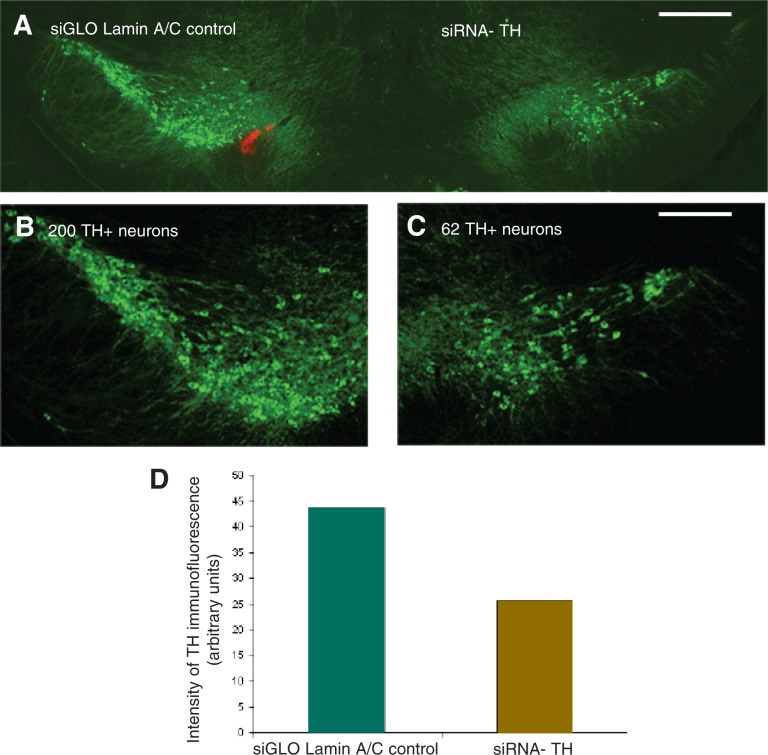
Effectiveness of siRNA injected into brain in reducing the production of the enzyme tyrosine hydroxylase (TH) in mouse brain cells. TH expression in the cells is represented by the green fluorescence. **A)** Comparison of the intensity of TH fluorescence in the side of the brain treated with a control siRNA (left) and in the side treated with TH-siRNA (right). The intensity of the fluorescence in the control side of the brain is higher than in the TH-siRNA–treated side of the brain (see D). **B)** and **C)** Comparison of the number of TH-expressing cells in the same brain section shown in A. The number of TH-expressing cells is higher in the control side B than in the TH-siRNA–treated side C. **D)** The intensity of the fluorescence is greater in the control side than in the TH-siRNA– treated side. NOTE: Scale bar in A equals 1mm and in B and C equals 0.5 mm.

To date, the siRNA approach has not been used directly in alcohol research. However, researchers are beginning to use this technology for investigating genes that may play a role in mediating alcohol’s effects on the brain. For example, some experiments are beginning to investigate the effects of a single injection of siRNA targeting the enzyme tyrosine hydroxylase (TH) into a certain region of the mouse brain. TH is an enzyme that mediates one step in the synthesis of the signaling molecule (i.e., neurotransmitter) dopamine, which in turn has a central role in mediating the rewarding and reinforcing effects of alcohol. Initial analyses found that a single injection of siRNA targeting TH mRNA reduced the number of neurons in which TH could be detected as well as the amounts of TH in those cells in which it still was expressed (see figure 11).

## Conclusion

siRNA is a novel approach that holds great scientific and therapeutic potential by allowing researchers to selectively reduce or eliminate the expression of individual genes. Some studies already have demonstrated the feasibility and utility of this approach. siRNAs hold promise for studies of brain function and currently are used to investigate potential roles of specific genes expressed in the brain in the acquisition and maintenance of alcohol preference. To date, the main shortfall of this approach is that silencing induced by a single application of siRNA is transient and generally lasts for less than a week following transfection. Additional studies therefore are focusing on modifying the route of administration of siRNAs in order to achieve longer-lasting effects. One of these approaches is discussed in the following article by Lasek and Heberlein (pp. 259–260).

## Footnotes

1 In some instances, siRNA also can have nonspecific effects that are not induced by its interaction with the specific target mRNA. In these cases, the siRNA molecules act non-specifically on a molecule called Toll-like receptor 3, which is an immunoregulatory “sensor” present on the cell surface. Thus, this nonspecific effect does not require the siRNA to enter the cell. Nevertheless, it can have profound consequences by activating the innate immune responses.
